# Efficacy and Safety of Ligation Combined With Sclerotherapy for Patients With Acute Esophageal Variceal Bleeding in Cirrhosis: A Meta-Analysis

**DOI:** 10.3389/fsurg.2021.664454

**Published:** 2021-06-09

**Authors:** Juan Su, Huilin Zhang, Maifang Ren, Yanan Xing, Yuefei Yin, Lihua Liu

**Affiliations:** ^1^Department of Gastrology Ward III, Xi'an International Medical Center Hospital, Xi'an, China; ^2^Department of Digestive Endoscopy and Treatment Center, Xi'an International Medical Center Hospital, Xi'an, China; ^3^Department of Gastrology Ward I, Xi'an International Medical Center Hospital, Xi'an, China

**Keywords:** esophagogastric variceal bleeding, endoscopic variceal ligation, endoscopic injection sclerotherapy, cirrhosis, meta- analysis

## Abstract

**Objective:** To evaluate the efficacy and safety of endoscopic variceal ligation + endoscopic injection sclerotherapy (EVL+EIS) to control acute variceal bleeding (AVB).

**Methods:** Online databases, including Web of Science, PubMed, the Cochrane Library, Chinese National Knowledge Infrastructure (CNKI), China Biology Medicine (CBM) disc, VIP, and Wanfang, were searched to identify the studies comparing the differences between EVB+EIS and EVB, EIS from the inception of the databases up to December 30, 2020. STATA 13.0 was used for the meta-analysis.

**Results:** A total of eight studies involving 595 patients (317 patients in the EVL group and 278 patients in the EVL+EIS group) were included. The results of the meta-analysis did not reveal any statistically significant differences in the efficacy of acute bleeding control (*P* = 0.981), overall rebleeding (*P* = 0.415), variceal eradication (*P* = 0.960), and overall mortality (*P* = 0.314), but a significant difference was noted in the overall complications (*P* = 0.01).

**Conclusion:** EVL is superior to the combination of EVL and EIS in safety, while no statistically significant differences were detected in efficacy. Further studies should be designed with a large sample size, multiple centers, and randomized controlled trials to assess both clinical interventions.

## Background

Esophagogastric variceal bleeding (EVB) is the most dangerous complication of decompensated cirrhosis (1). Most of the patients with liver cirrhosis have symptoms of esophagogastric varices, with an increase in the incidence by 7% per year (2). EVB is the main influencing factor for the increased mortality in patients with liver cirrhosis (3). The mortality of the first bleeding was about 20–30% if an active intervention was not carried out (4). Within 2 years after the first bleeding, the rebleeding rate and mortality increased significantly, which threatened the safety of patients (5). However, the secondary prevention of EVB in liver cirrhosis mainly includes endoscopic treatment, non-selective beta-blocker drugs (NSBBs), transjugular intrahepatic portosystemic shunt (TIPS), and surgical treatment (6); all these methods have limited curative effects. Although the evidence is not convincing, guidelines recommend the use of ligation and vasoactive drugs as first-line therapy for acute variceal bleeding (AVB) (7).

In the development of endoscopic therapy technology, sclerosing agent injection, tissue glue injection, vein ligation, and several other technical methods have emerged gradually to control acute bleeding and prevent rebleeding (8). Previous studies and meta-analyses have shown that vasoactive drugs and sclerotherapy are better than sclerotherapy alone (9). However, the clinical outcomes were not evaluated with respect to endoscopic variceal ligation (EVL) combined with endoscopic injection sclerotherapy (EIS). Thus, we conducted a meta-analysis to investigate the efficacy and safety of EVL+EIS to control AVB.

## Methods

### Inclusion and Exclusion Criteria

#### Inclusion Criteria

(1) Patients: Liver cirrhosis patients with AVB >18 years old. Among them, nationality and race. are not limited.
(2) Interventions: Clinical interventions are EVB combined with EIS, EVB, or EIS.
(3) Outcomes: Bleeding control rate, risk of overall rebleeding, rebleeding rate, overall mortality, and complications.
(4) Study design: Types of included studies are retrospective, prospective, and randomized controlled trials (RCTs).

#### Exclusion Criteria

(1) Patients with hepatocellular cell carcinoma or other malignancies.
(2) Publications based on animal experiments.
(3) Duplication, abstract, conference papers, and articles without detailed data were also excluded.

### Database Search Strategy

The online databases, including Web of Science, PubMed, the Cochrane Library, Chinese National Knowledge Infrastructure (CNKI), China Biology Medicine disc (CBM), VIP, and Wanfang, were searched, and the studies that compared the differences between EVB combined with EIS and EVB, EIS were identified from the inception of the databases up to December 30, 2020. Free terms and subject terms were combined, and the language was restricted to English and Chinese. The key search words were “endoscopic variceal ligation,” “endoscopic injection sclerotherapy,” “EVL,” “EIS,” “cirrhosis,” “esophageal variceal bleeding.”

### Data Extraction

Two researchers extracted the data from the studies independently. The information included the following: (1) General characteristics of the included studies: authors, country, study design, sample size, mean age, the main cause of cirrhosis, and Child–Pugh score; (2) Outcomes: efficacy of bleeding control, overall rebleeding rate, overall mortality, variceal eradication, and complications.

### Risk of Bias Assessment

The methodological quality and bias assessment were completed by two reviewers. The risk of bias was assessed using the Cochrane Collaboration tool, which rates seven items as high, low, or unclear for risk of bias (10). These items include random sequence generation, allocation concealment, blinding of participants and personnel, blinding of outcome assessment, incomplete outcome data, selective outcome reporting, and other potential sources of bias.

### Data Analysis

STATA 13.0 was used for the meta-analysis. χ^2^-test and *I*^2^-test are used to determine the heterogeneity among the studies. If *I*^2^ <50%, *P* > 0.1, there is no heterogeneity in the data analysis, and a fixed-effects model was used; if not, the random-effects model assessed the different causes of heterogeneity. Sensitivity analysis was carried out when the subgroup analysis was not satisfactory, and it was employed to evaluate the robustness of the main results.

## Results

### Characteristics of the Included Studies

**Figure 1:**
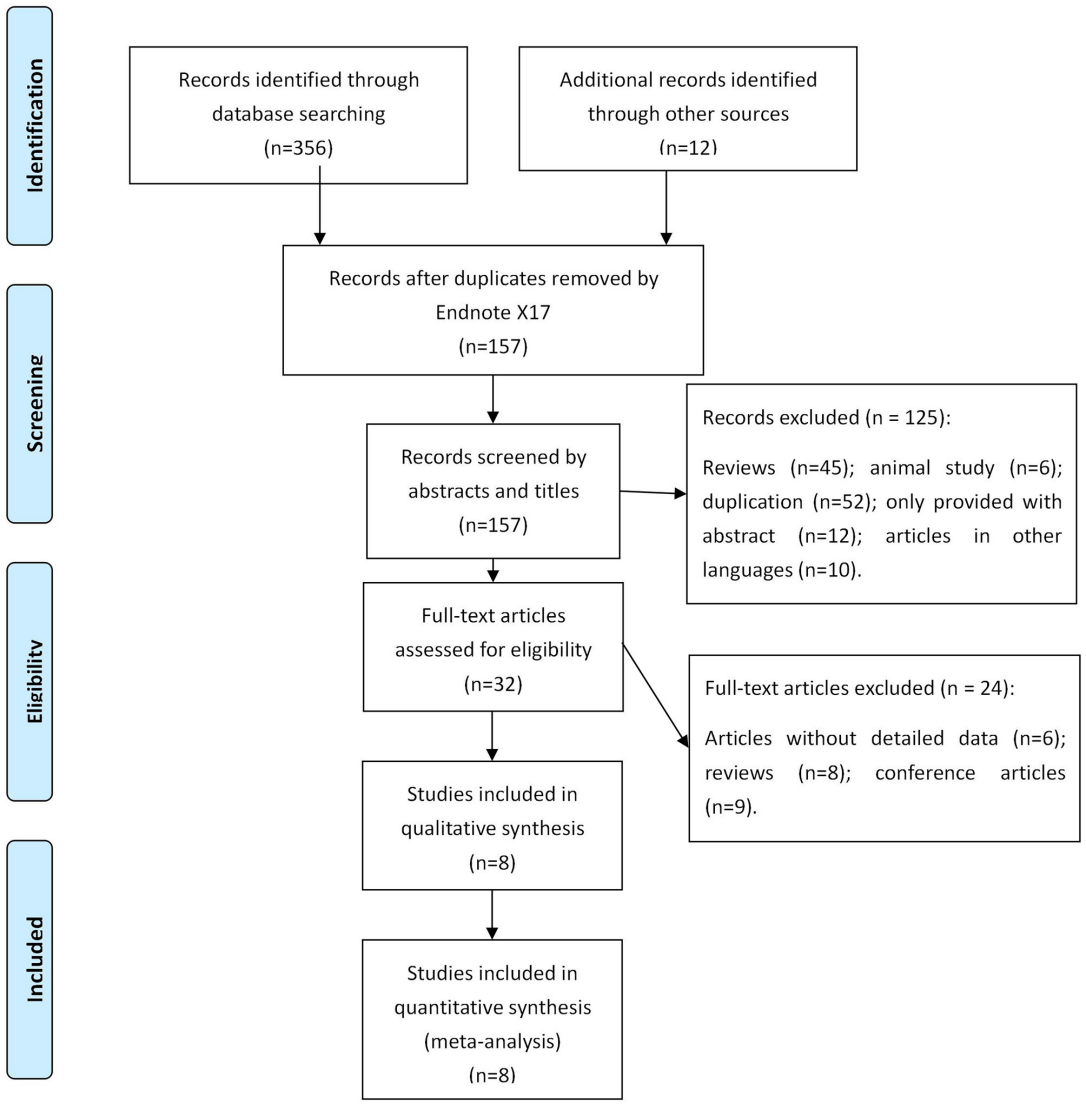
Schematic of literature selection.

A total of 368 records were searched in online databases. After assessing the titles and abstracts, 211 studies were identified as eligible citations. Full-text reading retrieved eight studies (11–18) involving 595 patients (317 patients in the EVL group and 278 patients in the EVL+EIS group) (Figure 1).

Among the eight included studies, three were from the USA, and five were designed as RCTs. The main courses of cirrhosis were hepatitis B virus (HBV), hepatitis C virus (HCV), and alcohol. The characteristics of the included studies are listed in Table 1.

**Table 1:** The characteristics of included studies.

**Study**	**Country**	**Study design**	**No. of patients (*****n*****)**		**Mean age**	**Male**	**Main cause of cirrhosis**		**Child–Pugh class C (*****n*****, %)**	
			**EVL**	**EVL+EIS**	**(Years)**	**(%)**	**EVL**	**EVL+EIS**	**EVL**	**EVL+EIS**
Laine et al. (11)	USA	RCT	20	21	47	73.2	Alcohol	Alcohol	9 (45.00)	9 (42.86)
Saeed et al. (12)	USA	RCT	25	22	53.1	91.5	Alcohol	Alcohol	15 (16.00)	9 (40.91)
Traif et al. (13)	Saudi Arabia	RCT	31	29	48.8	61.7	HCV	HCV	10 (32.26)	5 (17.24)
Djurdjevic et al. (14)	USA	Prospective study	51	52	55.6	61.2	Alcohol	Alcohol	12 (23.23)	10 (19.23)
Umehara et al. (15)	Japan	RCT	26	25	58.2	62.3	HBV	HBV	6 (23.07)	4 (16.00)
Harras et al. (16)	Egypt	Prospective study	50	50	48.9	46.9	HCV	HCV	4 (0.08)	2 (0.04)
Mansour et al. (17)	Egypt	RCT	60	60	NA	65	HCV	HCV	32 (53.33)	24 (40.00)
Zheng et al. (18)	China	Prospective study	54	19	55.2	65.4	HBV	HBV	14 (9.21)

*RCT, randomized controlled trail; HCV, Hepatitis C virus; HBV, Hepatitis B virus*.

**Figure 2:**
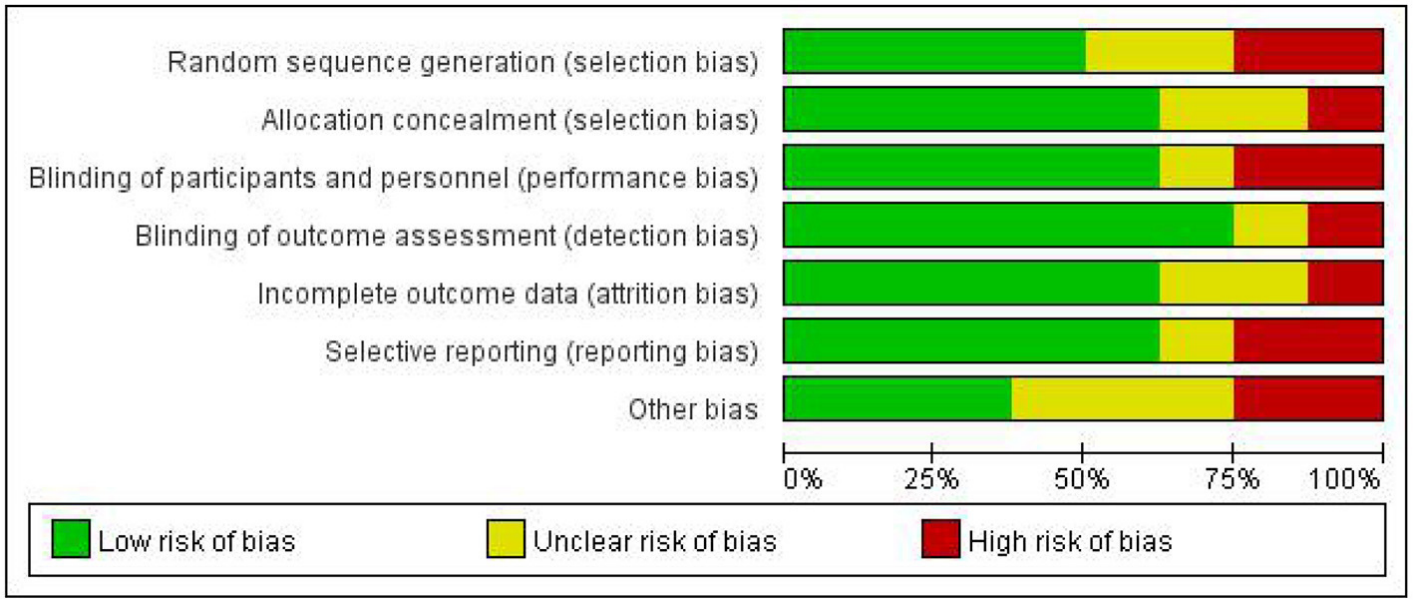
Summary of the assessment of risk of bias.

**Figure 3:**
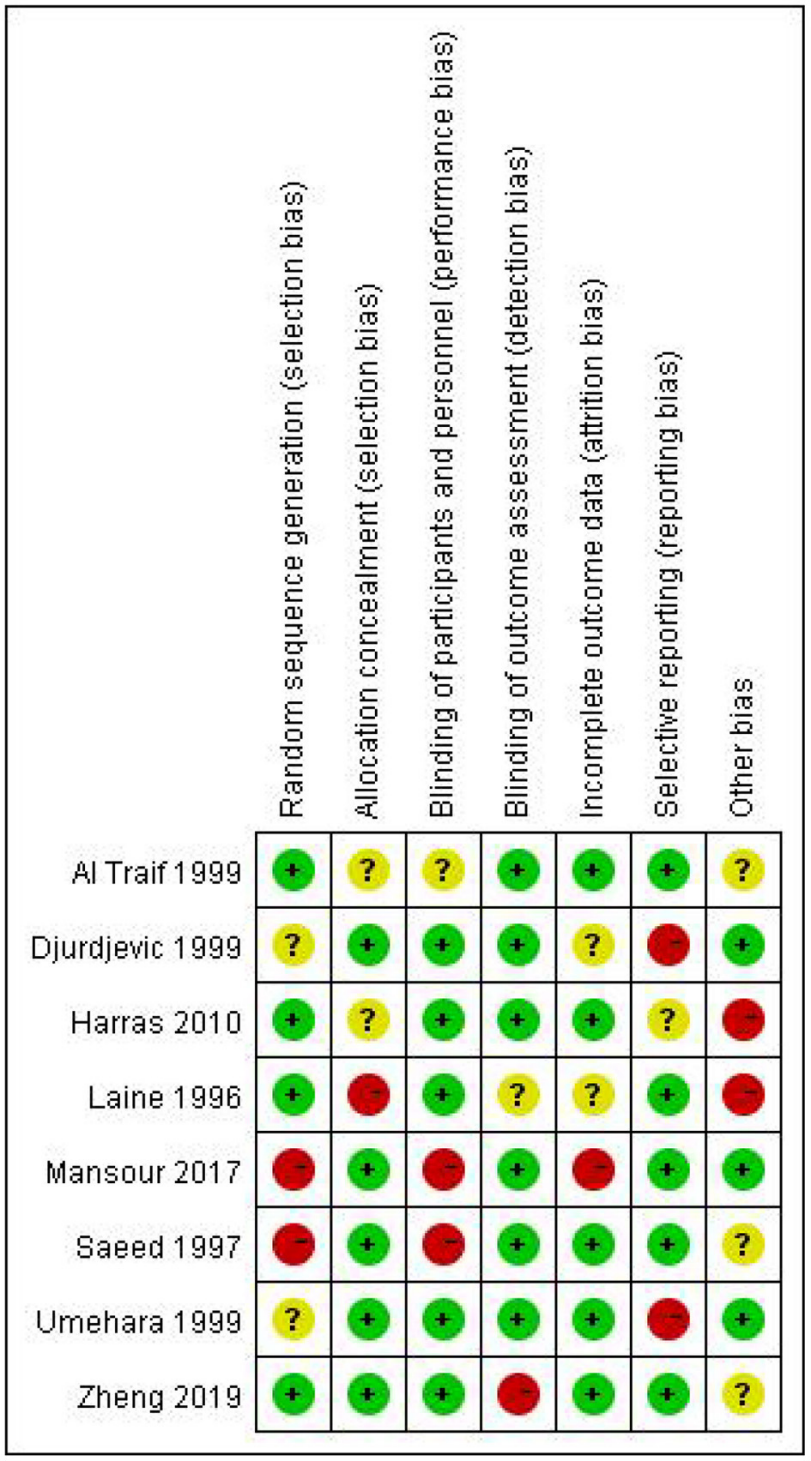
Assessment of risk of bias.

None of the included studies were assessed to have a low risk of bias in all the seven items of the Cochrane Collaboration tool (Figure 2). The majority of the studies were high risk for random sequence generation and for other sources of bias (Figure 3). Studies scored high risk for other sources of bias with respect to concerns, such as baseline differences and industry funding. Most of the studies had an unclear risk of bias for selective outcome reporting, and a few had registered protocols.

### Results of the Meta-Analysis

#### Efficacy of Acute Bleeding Control

**Figure 4:**
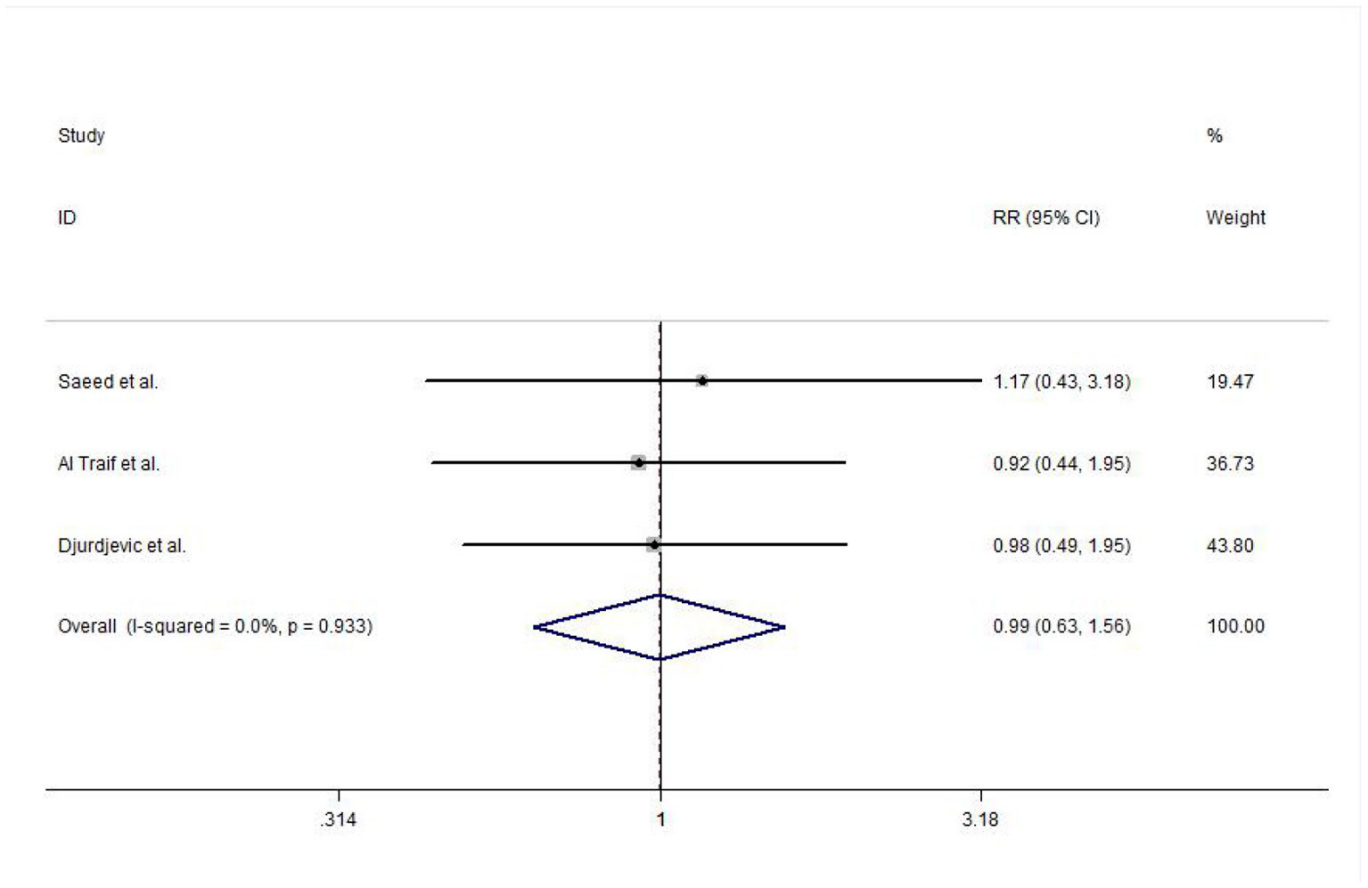
Forest plot of the meta-analysis comparing EVL and EVL+EIS with respect to the efficacy of acute bleeding control. EIS, endoscopic injection sclerotherapy; EVL, endoscopic variceal ligation.

In this meta-analysis, three studies reported the efficacy of acute bleeding control. No heterogeneity was detected between studies (*I*^2^ = 0.0%, *P* = 0.933), and the meta-analysis was conducted using a fixed-effects model. The results did not show any significant difference between EVL and EVL+EIS interventions (risk ratio (RR) = 0.99, 95% CI: 0.63–1.56, *P* = 0.981; Figure 4).

#### Overall Rebleeding

**Figure 5:**
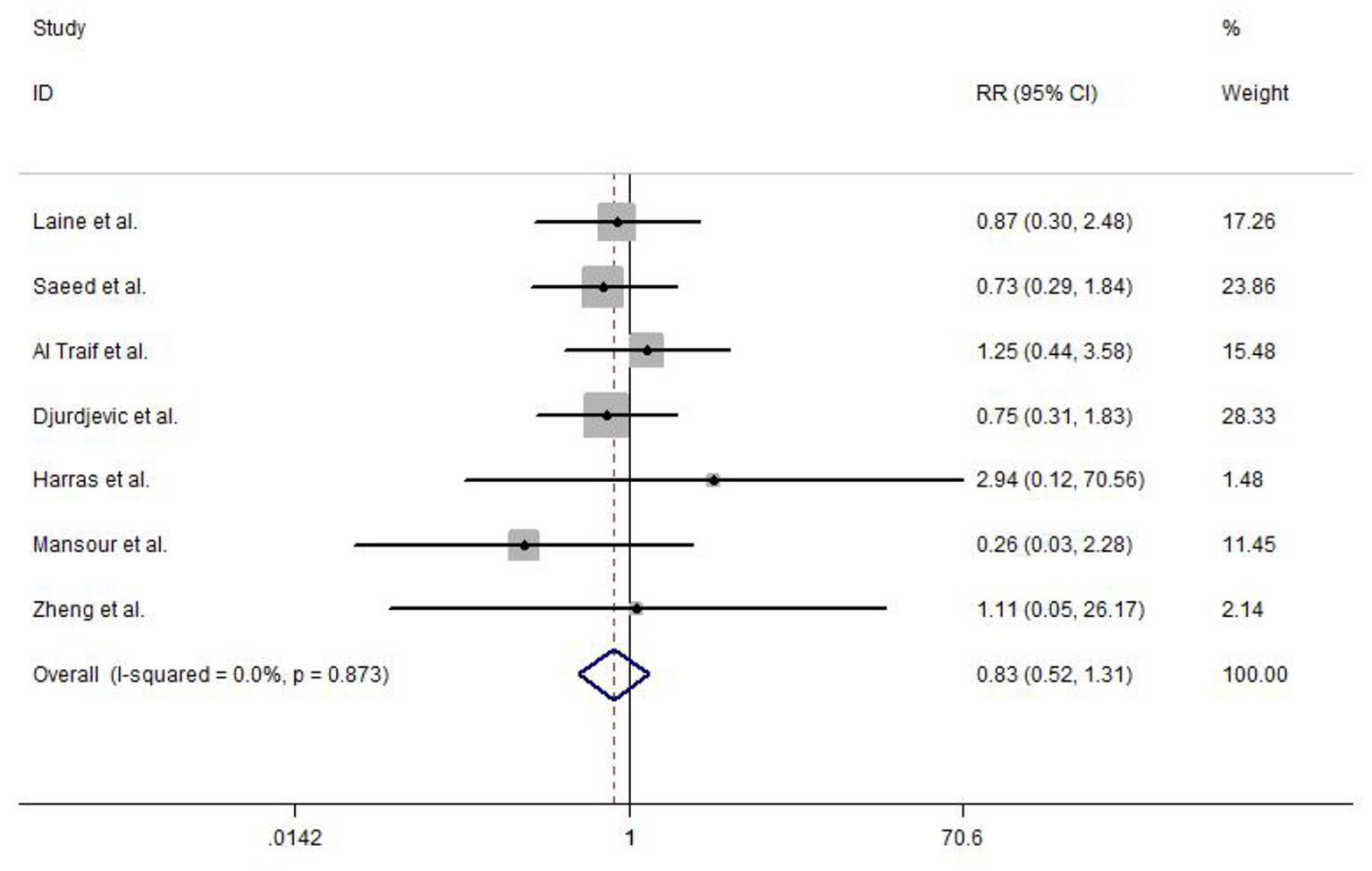
Forest plot of the meta-analysis comparing EVL and EVL+EIS in overall rebleeding. EIS, endoscopic injection sclerotherapy; EVL, endoscopic variceal ligation.

An overall rebleeding was reported in seven included studies, and no heterogeneity was observed between studies (*I*^2^ = 0.0%, *P* = 0.873). The meta-analysis was conducted using a fixed-effects model. No statistically significant difference was detected in EVL and EVL+EIS (RR = 0.83, 95% CI: 0.52–1.31, *P* = 0.415; Figure 5).

#### Variceal Eradication

**Figure 6:**
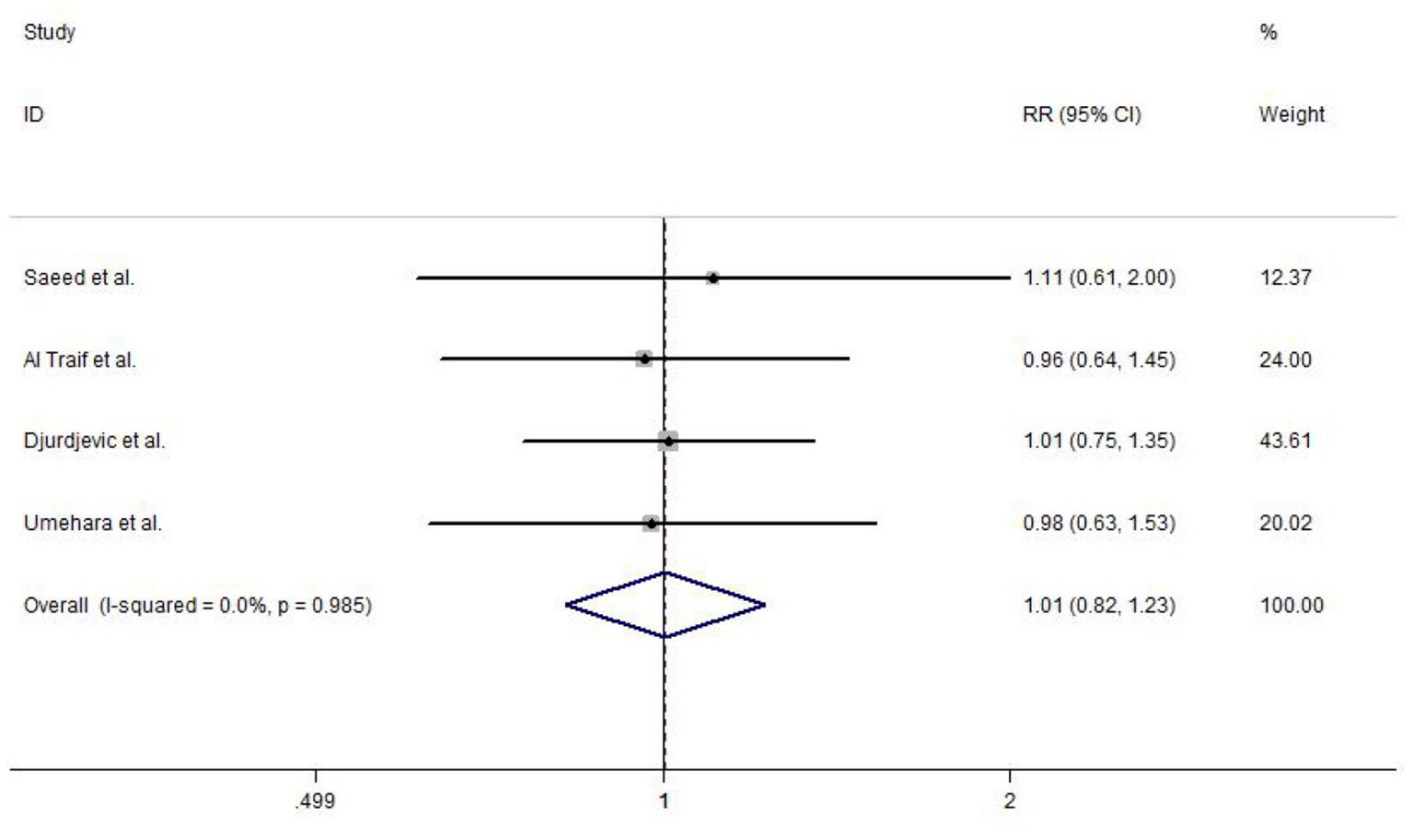
Forest plot of the meta-analysis comparing EVL and EVL+EIS in variceal eradication. EIS, endoscopic injection sclerotherapy; EVL, endoscopic variceal ligation.

Among the included studies, four reported variceal eradication. The meta-analysis using a fixed-effects model (study heterogeneity: *I*^2^ = 0.0%, *P* = 0.985) did not detect any statistically significant difference in EVL and EVL+EIS (RR = 1.01, 95% CI: 0.82–1.23, *P* = 0.960; Figure 6).

#### Overall Mortality

**Figure 7:**
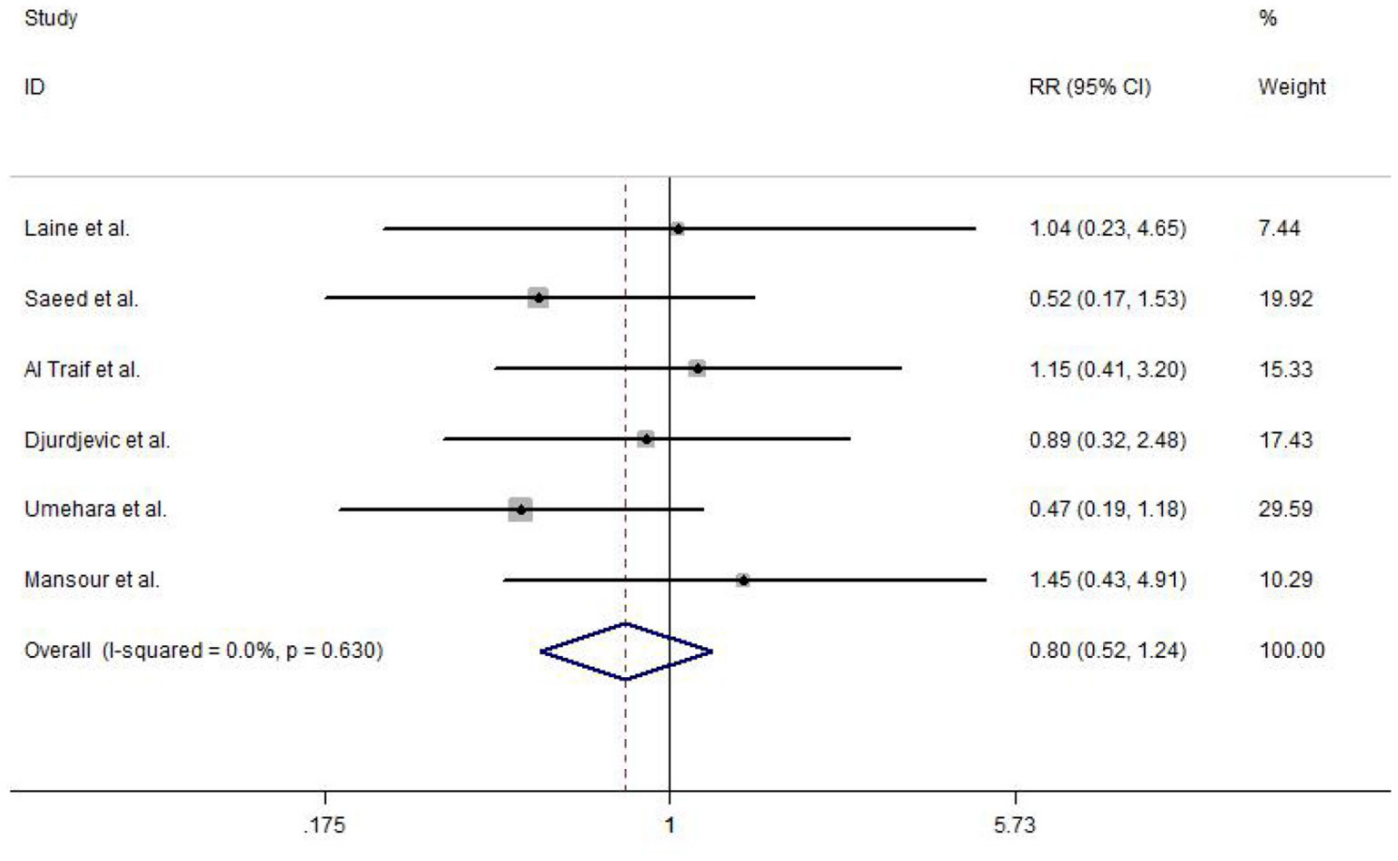
Forest plot of the meta-analysis comparing EVL and EVL+EIS in overall mortality. EIS, endoscopic injection sclerotherapy; EVL, endoscopic variceal ligation.

The overall mortality was reported in six included studies. No heterogeneity test was observed between studies (*I*^2^ = 0.0%, *P* = 0.630), and hence, a fixed-effects model was used to analyze the data. Strikingly, no statistically significant difference was detected in EVL and EVL+EIS (RR = 0.80, 95% CI: 0.52–1.24, *P* = 0.314; Figure 7).

#### Complications

**Figure 8:**
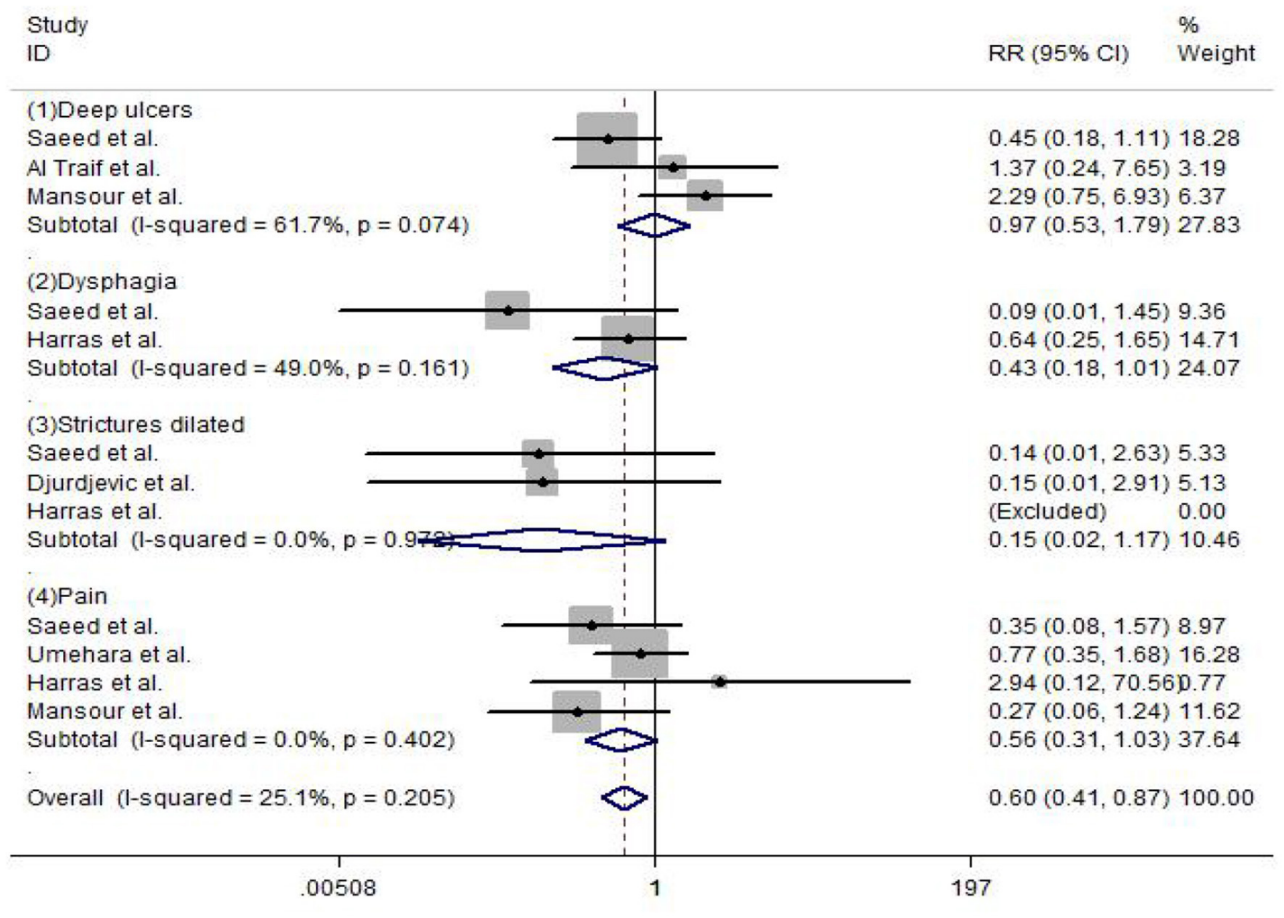
Forest plot of the meta-analysis comparing EVL and EVL+EIS in complications. EIS, endoscopic injection sclerotherapy; EVL, endoscopic variceal ligation.

Complications were reported in the included studies. The results of the meta-analysis show that deep ulcers (RR = 0.97, 95% CI: 0.53–1.79, *P* = 0.247), dysphagia (RR = 0.43, 95% CI: 0.18–1.01, *P* = 0.106), strictures dilated (RR = 0.15, 95% CI: 0.02–1.17, *P* = 0.353), and pain (RR = 0.56, 95% CI: 0.31–1.03, *P* = 0.124) did not show any significant difference between EVL and EVL+EIS, but the overall complication rate (RR = 0.60, 95% CI: 0.41–0.87, *P* = 0.01) had a statistically significant difference between EVL and EVL+EIS interventions (Figure 8).

## Discussion

EVB patients have a high risk of rebleeding and death after bleeding control (19). If the EVB patients do not receive secondary preventive treatment for 1–2 years, the rebleeding rate is elevated to about 60%, and the mortality rate is 33% (20). At present, EVL and EIS are indispensable in the endoscopic treatment of the secondary prevention of EVB. The basic goal of the treatment is to eradicate or reduce the degree of esophageal varices in order to reduce the recurrence rate and mortality (21). Patients with a history of EVB should be treated routinely by endoscopy, and patients with acute EVB should continue to receive corresponding endoscopic treatment after the termination of bleeding (22).

In EVL technology, the negative pressure at the front end of the endoscope is inhaled into the esophageal varices that are then ligated with a rubber ring in the transparent cap (7). The physical ligation blocks the blood supply of the varices, resulting in thrombosis, tissue necrosis, and ulcers, finally leaving healing scars for the treatment and elimination of varices (23). EIS refers to the injection of a sclerosing agent into the tissue of varicose vein or adjacent to varicose vein, which shows ischemia and necrosis in the tissue of varicose vein, and then produces fibrosis, to eliminate varicose veins (24). With the continuous development of endoscopic technology and the evolution of sclerosing agents, the clinical application of EVL and EVs is also evolving (25).

The present meta-analysis did not detect any statistically significant difference in the efficacy of acute bleeding control (RR = 0.99, 95% CI: 0.63–1.56, *P* = 0.981), overall rebleeding (RR = 0.83, 95% CI: 0.52–1.31, *P* = 0.415), variceal eradication (RR = 1.01, 95% CI: 0.82–1.23, *P* = 0.960), and overall mortality (RR = 0.80, 95% CI: 0.52–1.24, *P* = 0.314), but a significant difference was observed in the overall complications (RR = 0.60, 95% CI: 0.41–0.87, *P* = 0.01). The main complications of EVL include chest pain or discomfort, dysphagia or pain, and erosion or ulcer at the ligation site, infection, or bacteremia (26). Rubber bands falling off and sliding can also form ulcers and after rebleeding (27). Compared to EVL alone, the effect of EIS combined with EVL varies in different studies. In patients with active bleeding, EVL uses ligation device, which limits the intraoperative field of vision, raising the technical requirements of endoscopic operators (28).

Due to various conditions, the present meta-analysis has some limitations. Firstly, the included studies were from different countries. Secondly, the frequency of follow-up and the total duration of follow-up were also incompatible. Thirdly, some disparities in medical technology and medical facilities were observed in the included literature. Therefore, EVL and EVs may show similar results in the treatment of esophageal variceal bleeding.

In conclusion, EVL is superior to the combination of EVL and EIS in safety, while no significant differences were noted in efficacy. Nonetheless, further studies should be designed based on a large sample size, multiple centers, RCTs to substantiate these two clinical interventions.

## Data Availability Statement

The original contributions presented in the study are included in the article/supplementary material, further inquiries can be directed to the corresponding author/s.

## Author Contributions

JS and HZ contributed to the conceptualization, project administration, and writing and review. JS and MR contributed to the data curation. YX and YY contributed to the data analysis. JS, HZ, and LL contributed to the methodology. HZ and MR contributed resources. YX and LL contributed the software. HZ contributed to the supervision. All authors contributed to the article and approved the submitted version.

## Conflict of Interest

The authors declare that the research was conducted in the absence of any commercial or financial relationships that could be construed as a potential conflict of interest.
